# Selenium-containing small molecules

**DOI:** 10.1038/s42004-022-00756-7

**Published:** 2022-10-28

**Authors:** Huijuan Guo

**Affiliations:** Communications Chemistry, https://www.nature.com/commschem

## Abstract

The role of selenium in proteins and nucleic acids is well-established, but the pathway for incorporating selenium into small molecules remains unknown. Now, using bioinformatics tools, a selenium-incorporating gene cluster is discovered from immense genomic data, leading to the identification of selenoneine from microorganisms and the elucidation of a selenium incorporation pathway.

Selenium is known to be a dose-dependent ‘essential poison’ trace element in diverse organisms. During evolution, organisms can convert inorganic forms of selenium (sodium selenite) from the environment into organic forms, such as selenoproteins and selenonucleic acids via selenocysteine and 2-selenouridine, respectively. These selenobiomolecules have important biological functions in various redox process^1^ Their biosynthesis involves a *sel* gene cluster containing three Se-specific enzymes: SelD catalyzes selenophosphate formation from selenide, SelA guides selenocysteine formation and SelU leads to 2-selenouridine. Selenoneine, an ergothioneine congener by replacing sulfur with selenium, is so far the only Se-containing small molecule reported from the tissue of marine animals, but its biosynthetic pathway is still poorly understood. Now, Seyedsayamdost and colleagues from Princeton University in the US report the discovery of microorganism-derived selenoneine and elucidate a widespread selenium-incorporation pathway and the molecular function of key enzymes for C-Se bond formation (10.1038/s41586-022-05174-2)^2^.

To explore Se-containing small molecules in microorganisms, the team used state-of-the art OMICs techniques, combining genomics and metabolomics. By using a bioinformatics search against the NCBI database, they searched for genes overlapping with *selD*, the protein product of which forms selenophosphate (SeP). They found hundreds of gene clusters, the *sen* cluster, encoding *selD* and at least two genes of unknown function. They then examined microorganisms that encode the *sen* locus and detected the production of selenoneine by HRMS and NMR techniques. The researchers subsequently reconstituted the entire pathway in vitro. In doing so, they elucidated the biosynthetic pathway for selenoneine and characterized the molecular function of key enzymes in the *sen* cluster: SenC transforms sodium selenite to SeP, then SenB synthesizes a selenosugar, which is used by SenA as a Se-source to transform hercynine into selenoneine (Fig. 1).

**Fig. 1:**
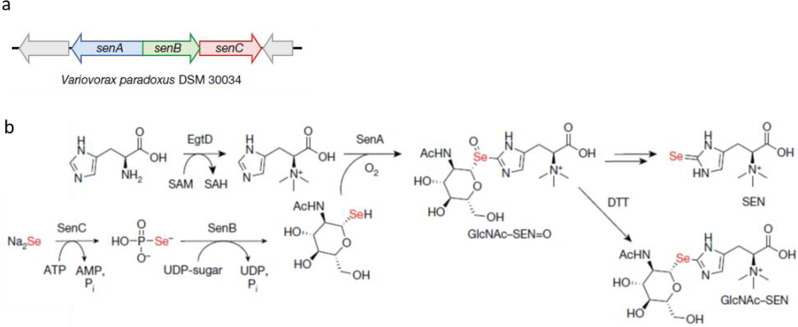
Elucidation of a selenium incorporation pathway in microorganisms. **a** The *sen* gene cluster from the ß-proteobacterium *V. paradoxus* DSM 30034 and **b** the biosynthetic pathway of selenoneine (SEN). © The Author(s), under exclusive licence to Springer Nature Limited 2022.

“The biggest challenge was figuring out what reactions the enzymes in this pathway actually catalyze.” comments Seyedsayamdost. “One enzyme, which we named selenosugar synthase (SenB) had no significant homology to any known proteins. We speculated what reaction it may catalyze based on protein structure and homology modeling and ultimately, using iterative computational analysis and biochemical experiments, elucidated its reaction.”

This pioneering work is likely to serve as a stepping stone for future research on selenium-related catalytic mechanisms and related potential biological and synthetic utilities. “The key implication of our work is that there exists a selenometabolome. We found the first pathway, but there are likely others and one of our next goals is to identify additional microbial selenometabolites. The reaction mechanisms of SenA and SenB are intriguing and of interest as well.”, concludes Seyedsayamdost.
